# Safety of adult medical male circumcision performed by non-physician clinicians in Kenya: a prospective cohort study

**DOI:** 10.9745/GHSP-D-13-00120

**Published:** 2014-01-09

**Authors:** Vera Frajzyngier, George Odingo, Mark Barone, Paul Perchal, Melinda Pavin

**Affiliations:** a EngenderHealth, New York, NY, USA; b EngenderHealth, Nairobi, Kenya

## Abstract

**Background:**

Male circumcision reduces the risk of heterosexually acquired HIV infection in men by approximately 60%. The Government of Kenya is rolling out voluntary medical male circumcision (VMMC) services, but struggles with health worker shortages, particularly with physician shortages.

**Objective:**

To evaluate the safety of male circumcision performed by non-physician clinicians in Kenya.

**Methods:**

Between December 2009 and December 2010, we conducted a prospective study of VMMC procedures performed by 15 nurses and 11 clinical officers, all trained to competence, in 11 public health facilities in Nyanza Province, Kenya. Providers reported surgical complications and adverse events (AEs), based on standardized definitions, immediately after the procedure and at 7 days and 60 days post-circumcision. We also assessed clients' satisfaction with the circumcision at 60 days.

**Results:**

We recruited 2,244 men and boys, ages 13–54. The retention rate was high, with 2,192 participants (98%) returning for the 7-day follow-up visit and 1,845 (82%) for the 60-day visit. There was no difference in rates of moderate and severe AEs between participants whose circumcision was performed by a nurse (2.1%) or a clinical officer (1.9%) at 7 days post-circumcision. The most common AE was excess swelling (1.1%). Risk factors associated with an AE at 7 days post-circumcision included being employed and participant age ≥ 18 years. Participants circumcised by a provider with ≥ 6 years of professional experience were less likely to have an AE. Nearly all participants reported being satisfied with their circumcision at the 60-day follow-up visit.

**Conclusions:**

Trained nurses and clinical officers provided safe VMMC in Nyanza Province, Kenya. AE rates in this study were similar to those reported in typical service-delivery settings. These results add to the current body of evidence suggesting that trained non-physicians can provide safe medical male circumcision, thereby facilitating increased availability and access to circumcision services.

## Abstract (precis)

Trained, experienced nurses and clinical officers provided safe voluntary medical male circumcision (VMMC) in public health facilities in Nyanza Province, Kenya, as evidenced by the low 2% adverse event rate (most commonly, excess swelling). Task shifting for male circumcision can improve access to quality VMMC services.

## INTRODUCTION

In 2007, based on results from 3 randomized controlled studies,^1^^–^^3^ the World Health Organization (WHO) and the Joint United Nations Programme on HIV/AIDS (UNAIDS) determined there was compelling evidence that male circumcision reduces the risk of heterosexually acquired HIV infection in men by approximately 60%.^4^^,^^5^ Later studies found that this protective effect against HIV is sustained over extended follow up (up to 6 years in the studies).^6^^,^^7^ As such, in countries with high HIV prevalence, a generalized heterosexual HIV epidemic, and low rates of male circumcision, WHO and UNAIDS recommend safe, voluntary circumcision for adult men as one important component of a comprehensive strategy to prevent HIV.^4^

Male circumcision reduces the risk of heterosexually acquired HIV infection in men by about 60%.

Nyanza Province in Kenya meets these criteria for voluntary medical male circumcision (VMMC) services. In 2008, the overall HIV prevalence rate in Kenya was 5%, with approximately 83% of men circumcised, while in Nyanza Province, HIV prevalence was 12%, with only 46% of men circumcised.^8^ Luos, an ethnic group of Kenya concentrated in Nyanza Province,^9^ had the highest rate of HIV (20%), with only 22% of Luo men circumcised.^8^

In 2009, the Kenyan Ministry of Health rolled out a policy for adult male circumcision in response to the WHO/UNAIDS recommendation. The goal was to provide a framework to ensure safe, accessible, and sustainable male circumcision services.^10^^,^^11^

Although Kenya is committed to rolling out VMMC services, availability of health care providers, particularly physicians, is limited.^10^^–^^12^ Clinical officers were initially trained as the primary provider of male circumcision services. These are mid-level, non-physician clinicians, similar to physician assistants, whose preservice training focuses on clinical skills, often including minor surgeries. In 2009, the government approved nurses to also perform the procedure.^13^

Task shifting to include nurses as circumcision providers may be one short-term strategy for addressing the gap in human resources, especially in rural communities where there tend to be fewer clinical officers and physicians. Indeed, by the end of 2011, 10 of the 14 sub-Saharan African countries where scale up of VMMC services was deemed to be a priority had implemented task shifting, and 4 of these countries had explicit task-shifting policies.^14^

Task shifting for male circumcision may help address the health worker shortage problem in many countries.

Ensuring safety of the male circumcision procedure is of critical importance, particularly when implemented as part of mass campaigns or when conducted by lower-level cadres. Adverse event (AE) rates in controlled trials have ranged from 1.5% in Kisumu, Kenya,^2^ to 3.6% in Rakai, Uganda,^3^ and 3.8% in Orange Farm, South Africa.^1^ Adverse events reported in these studies included pain,^1^ bleeding,^1^^,^^2^ swelling,^1^^,^^2^ and infection,^1^^–^^3^ among others. A recent systematic review and meta-analysis of circumcisions conducted in the context of task shifting or task sharing found AE rates ranging from 0.70% (95% confidence interval [CI] = 0.44%–1.02%) to 37.36% (95% CI = 27.54%–47.72%), with an overall pooled proportion of 2.31% (95% CI = 1.46%–3.16%).^15^

This article reports findings from a study conducted by EngenderHealth, under the umbrella of the Male Circumcision Consortium, to assess the safety of circumcisions performed by non-physician clinicians (clinical officers and nurses) as part of routine service provision, to better inform the Government of Kenya and the Ministry of Health regarding the feasibility of rolling out circumcision services by non-physicians. The Male Circumcision Consortium worked closely with the Kenyan Ministry of Health to help inform the provision and scale up of safe VMMC services.

The objectives of the study were:

1. To determine the proportion of men experiencing AEs at surgery and at 7 days and 60 days post-circumcision, by cadre
2. To evaluate risk factors associated with AEs post-circumcision
3. To assess client satisfaction with circumcision at 60 days post-surgery

## METHODS

### Study Participants

Between December 2009 and December 2010, men seeking circumcision at 11 health care facilities (6 hospitals, 4 health centers, and 1 dispensary) in Nyanza Province, Kenya, that provided integrated VMMC services were recruited for the study. Inclusion criteria included:

- 13–54 years old (reflecting ages of the boys and men seeking circumcision services at the sites)
- In good general health (per clinician assessment)
- Able to understand the study procedures and requirements
- Willing to return to the health care facility for follow up 7 days and 60 days after the procedure
- No contraindications to circumcision per the WHO male circumcision manual^16^

Informed consent was obtained from all participants in their local language prior to participation in the study. Minors (those under age 18) were asked to sign an assent form with consent provided by a parent.

### Study Procedures

Circumcision procedures and all associated VMMC services were carried out in accordance with the “Policy on Male Circumcision in Kenya.”^10^^,^^11^ All providers in this study were trained on male circumcision techniques, through a training program in Nyanza, based on the WHO/UNAIDS “Manual for Male Circumcision Under Local Anaesthesia.”^16^ The manual covers screening, preparation for surgery, surgical procedures, postoperative care, infection control, management of complications, and follow-up care. All providers were trained to competence in male circumcision per Kenya's policy on VMMC—that is, competency is achieved after following a formal training based on the WHO/UNAIDS manual and after performing 40 circumcisions under supervision; proficiency is achieved when a provider performs about 200 circumcisions.

All non-physicians in this study were trained on male circumcision techniques to the level of competence.

The facilities involved in the study had adequate equipment and supplies as well space where confidential counseling and safe and hygienic procedures could be provided. Per Kenya's policy on male circumcision, in addition to the circumcision procedure, all study sites provided comprehensive HIV-prevention services, including:

- Behavior change communication and risk reduction counseling about safer sex practices
- Provision of male condoms and education about the need to use condoms consistently and correctly
- HIV testing and counseling
- Counseling and services for sexually transmitted infections (STIs)
- HIV care and treatment services, as appropriate

Providers were asked to complete a questionnaire about their demographic, educational, and professional profiles. Using standardized case report forms, trained research assistants and male circumcision providers collected data on clients' sociodemographic background, details of the preoperative clinical examination, procedures used during the circumcision, and surgical complications/AEs experienced immediately after the circumcision, as well as 7 days and 60 days following the procedure.

All clients were interviewed about satisfaction with their circumcision 7 days and 60 days following the procedure. A 7-day follow-up visit is standard in the Kenya VMMC program. A follow-up visit at 60 days was included in the study to evaluate AEs that may not have been apparent until healing was advanced or complete (such as torsion, excessive skin removal) and to gather information on client satisfaction after healing was complete.

Participants were given a modest travel allowance (the equivalent of approximately US$3.00) at their 7-day and 60-day follow-up visits to enhance retention throughout the study.

The study was approved by the Institutional Review Board at the Kenya Medical Research Institute as well as the Protection of Human Subjects Committee at FHI 360.

### Study Measures

We defined AEs based on standardized definitions outlined in the WHO male circumcision manual^16^ and listed on Kenya's VMMC Adverse Events reporting form,^17^ as well as based on definitions used by other researchers.^2^^,^^3^ Each post-discharge AE was graded on level of severity (mild, moderate, or severe) by the male circumcision provider examining the study participant. We then convened a panel of research, HIV, and clinical experts to review the AEs in order to avoid inclusion of events that were not considered causally associated with the circumcision procedure or were within normal limits. We report only moderate and severe AE rates because most mild AEs are generally considered within normal limits; this has become the standard for reporting clinical outcomes of male circumcision studies.^18^^–^^22^

Adverse event reports were based on standardized definitions.

We examined both provider- and client-related factors as possible risk factors associated with AEs.

Provider factors included:

- Cadre (clinical officer, nurse)
- Sex
- Years of professional experience (< 6, ≥ 6)
- Number of procedures conducted during the course of the study (< 100, ≥ 100)
- Duration of the surgery (continuous variable, as well as categorized: < 19 minutes, 19–23 minutes, > 23 minutes)

Client factors included:

- Age (13–17 years, 18–54 years)
- Education (no secondary, at least some secondary)
- Marital status (single, married or living with partner)
- Employment status (employed, unemployed, student)
- Distance traveled to clinic (≤ 5 km, > 5 km)
- Mode of transportation to the health facility (walk, car or bus, motorcycle or bicycle, other)
- HIV status (negative, positive)

At 7 days post-circumcision, we asked men about resumption of routine activities, and at 60 days post-circumcision, we assessed men's satisfaction with their circumcision through responses to the following interview questions:

- Would you recommend male circumcision to others?
- How satisfied are you with the circumcision? (very satisfied, somewhat satisfied, very dissatisfied, somewhat dissatisfied)
- How satisfied is your partner with the circumcision? (very satisfied, somewhat satisfied, very dissatisfied, somewhat dissatisfied)

### Statistical Analysis

We calculated the proportion of men who experienced an AE associated with the circumcision prior to discharge and at 7 days and 60 days post-circumcision. We compared provider and client characteristics of those who presented with an AE at 7 days post-circumcision with those who did not by calculating odds ratios (ORs) and corresponding 95% confidence intervals. Odds ratios were derived using generalized estimating equations (GEE) to account for clustering of client outcomes within facilities.

Variables eligible for inclusion in multivariate analysis were conceptually associated with risk of AEs and statistically associated (*P* < .20) with the procedure in bivariate analysis. Adjusted ORs were also generated using GEE, accounting for clustering by facility. No AEs were reported when men were discharged following their circumcision, and few AEs were reported at 60 days post-circumcision, which precluded us from evaluating risk factors for AE at those points in time.

## RESULTS

### Provider Characteristics

A total of 26 trained circumcision providers (15 nurses and 11 clinical officers) performed VMMC on study participants. The number of procedures performed by nurses ranged from 5 to 206, and by clinical officers, from 2 to 259. All the clinical officers were male; 10 of the nurses were male and 5 female. On average, nurses were approximately 5 years older than clinical officers (average age of nurses, 39.3 years; clinical officers, 34.6 years).

Nurses worked, on average, about 4 more years in their profession than clinical officers (nurses, 13.7 years; clinical officers, 10.1 years). Somewhat before the study began, nurses' official scope of work did not include minor surgeries including male circumcision. However, they reported, on average, roughly the same number of years' experience performing minor surgeries as clinical officers (nurses, 10.1 years; clinical officers, 9.9 years) and male circumcisions (nurses, 4.3 years; clinical officers, 5.3 years).

In this study, clinical officers performed 818 (36%) circumcisions and the nurses, 1,426 (64%). On average, nurses conducted the surgery in slightly less time than clinical officers (mean, 22.4 minutes versus 24.5 minutes, respectively).

### Participant Characteristics

The study recruited 2,244 men and boys. Most study participants were ethnically Luo (98%) and were Christian (96%) (Table 1). Mean age was 22.7 years. The majority (72%) were single (not married, divorced, or widowed, or not living with a partner). Most men had at least some primary education (98%), and over 70% had at least some secondary education. Most of the participants were either students (42%) or employed (39%). In general, the study participants lived relatively close to the health care facility where they had their circumcision performed, traveling on average about 5.5 kilometers, and 58% walked to the clinic. About 6% reported they had HIV infection, and about 3% reported they had a sexual partner (wife, primary partner, or any other partner) who had HIV infection. According to the participant-reported data, among the men who had HIV infection, one-third had a discordant partner. Of the 2,244 men who were circumcised during the study, 2,192 (98%) returned for the 7-day follow-up visit, and 1,845 (82%) returned for the 60-day follow-up visit.

**TABLE 1.** Sociodemographic Characteristics of Study Participants (N = 2,244)

**Characteristics**	
Age, y, n (%)	
13–17	517 (23.0)
18–25	1,137 (50.7)
26–35	440 (19.6)
36–54	150 (6.7)
Age, y, mean (SD)	22.7 (7.4)
Ethnic group, n (%)	
Luo	2,191 (97.6)
Other	53 (2.4)
Religion, n (%)	
Christian	2,151 (95.9)
Muslim	18 (0.8)
Other	75 (3.3)
Marital status, n (%)	
Single	1,618 (72.1)
Married or living with partner	606 (27.0)
Unknown	20 (0.9)
Education, n (%)	
No secondary education	656 (29.2)
At least some secondary education	1,588 (70.8)
Employment status, n (%)	
Unemployed	401 (17.8)
Student	950 (42.4)
Employed	883 (39.3)
Unknown	10 (0.4)
Distance to clinic, km, mean (SD)	5.5 (6.5)
Mode of transportation to clinic, n (%)	
Walking	1,274 (57.7)
Car or bus	389 (17.6)
Motorcycle or bicycle	482 (21.8)
Other/unknown	99 (4.4)
HIV positive,^a^ n (%)	
Self	131 (5.8)
Partner (primary or other)	57 (2.5)

Abbreviations: SD, standard deviation.

a Self-reported by participants.

### Participants Experiencing an Adverse Event

No study participant experienced surgical complications or immediate postoperative AEs.

At 7 days post-circumcision, 2.0% of the men experienced a moderate or severe AE; the rates were similar between nurses and clinical officers (2.1% vs. 1.9%, respectively) (Table 2). The most common AE 7 days post-circumcision was excess swelling (about 1% among both nurses and clinical officers). There were no severe AEs, other than 2 cases of severe pain, as self-reported by men.

At 7 days post-circumcision, only 2.0% of the men experienced either moderate or severe adverse events.

**TABLE 2.** Adverse Events at 7 Days and 60 Days Post-Circumcision, by Type of Provider and Severity

	**7 days**			**60 days**		
**Adverse Event**	**Clinical Officer**	**Nurse**	**Total**	**Clinical Officer**	**Nurse**	**Total**
	**(n = 807)**	**(n = 1384)**	**(n = 2191)**	**(n = 694)**	**(n = 1151)**	**(n = 1845)**
Any moderate or severe AE	15 (1.9)	29 (2.1)	44 (2.0)	2 (0.29)	1 (0.09)	3 (0.16)
Pain						
Moderate	…	…	…	…	1 (0.07)	1 (0.05)
Severe	1 (0.12)	1 (0.07)	2 (0.09)	…	…	…
Infection						
Moderate	…	4 (0.29)	4 (0.18)	…	…	…
Severe	…	…	…	…	…	…
Excess swelling						
Moderate	8 (0.99)	15 (1.1)	23 (1.1)	…	…	…
Severe	…	…	…	…	…	…
Bleeding						
Moderate	1 (0.12)	…	1 (0.05)	…	…	…
Severe	…	…	…	…	…	…
Problems with appearance						
Moderate	2 (0.25)	5 (0.36)	7 (0.32)	1 (0.12)	…	1 (0.05)
Severe	…	…	…	…	…	…
Hematoma						
Moderate	2 (0.25)	1 (0.07)	3 (0.14)	…	…	…
Severe	…	…	…	…	…	…
Delayed wound healing						
Moderate	…	3 (0.22)	3 (0.14)	…	…	…
Severe	…	…	…	…	…	…
Injury to penis						
Moderate	…	…	…	1 (0.12)	…	1 (0.05)
Severe	…	…	…	…	…	…
Insufficient skin removed						
Moderate	1 (0.12)	…	1 (0.05)	…	…	…
Severe	…	…	…	…	…	…

Abbreviations: AE, adverse event.

Data are shown as n (%).

Men 18 years and older were significantly more likely than boys under 18 years old to experience an AE at 7 days post-circumcision. Specifically, compared with younger participants, men 18 years and older were significantly more likely to experience bleeding (0.5% versus 0%) and swelling (1.3% versus 0.2%) at the 7-day follow-up visit.

At 60 days post-circumcision very few men (0.2%) experienced moderate AEs, and no men had a severe AE (Table 2). There was no difference in AEs at 60 days between circumcisions performed by nurses and clinical officers.

At 60 days post-circumcision, very few men experienced moderate adverse events, and none had severe events.

At 7 days following their circumcision, 89% of the men reported they had resumed work, and 76% had resumed their leisure activities. Among those who had not resumed *work* activities by this time, 28% said they had too much pain or discomfort to do so. Among the men who had not resumed *leisure* activities by 7 days, 10% reported they had not done so due to pain and discomfort.

### Factors Associated With Adverse Events

In bivariate analyses, client age of 18 years and older was predictive of experiencing a moderate or severe AE, while students were less likely to experience a moderate or severe AE. Provider professional experience 6 years or higher was also associated with a lower likelihood of a participant experiencing a moderate or severe AE (*P* < .05) (Table 3).

**TABLE 3.** Risk Factors for Moderate/Severe Adverse Events at 7 Days Post-Circumcision

	**No. of AEs/Total**	**Unadjusted OR^a^ (95% CI)**	**Adjusted OR^b^ (95% CI)**
**Provider Characteristics**			
Sex			
Male	30/1833	Reference	…
Female	4/358	1.03 (0.40, 2.65)	…
Cadre			
Clinical officer	13/807	Reference	…
Nurse	21/1384	1.04 (0.42, 2.60)	…
Professional experience, y			
< 6	21/808	Reference	…
≥ 6	13/1383	0.45 (0.17, 1.17)	**0.39**^c^ **(0.17, 0.89)**
Procedure duration, min			
< 19	3/526	0.22 (0.01, 5.9)	…
19–23	13/689	0.77 (0.36, 1.63)	…
> 23	18/961	Reference	…
No. of procedures conducted during the study			
< 100	14/554	Reference	…
≥100	20/1637	0.64 (0.31, 1.32)	…
**Client Characteristics**			
Age, y			
13–17	2/505	Reference	…
18–54	32/1686	5.59 (1.48, 21.1)	**3.75**^c^ (**1.92, 7.33)**
Education			
No secondary education	13/626	Reference	…
At least some secondary education	21/1550	0.63 (0.35, 1.12)	0.61 (0.34,1.11)
Employment status			
Unemployed	4/395	Reference	…
Employed	21/867	1.27 (0.86, 1.89)	**2.41**^c^ (**1.06, 5.51)**
Student	9/919	0.65 (0.43, 0.97)	1.34 (0.76, 2.53)
Marital status			
Single	22/1572	Reference	…
Married or living with partner	12/599	1.38 (0.52 3.62)	…
Distance traveled, km			
≤ 5	16/1143	Reference	…
> 5	7/491	1.05 (0.46, 2.38)	…
Mode of transportation to the facility			
Walking	12/1246	Reference	…
Car or bus	10/379	2.66 (0.98, 7.2)	2.52 (0.94, 6.72)
Motorcycle or bicycle	9/474	1.99 (0.86, 4.59)	1.56 (0.78, 3.12)
Other	1/56	2.38 (0.62, 9.11)	1.56 (0.37, 6.54)
HIV status			
Negative	26/1775	Reference	…
Positive	4/130	2.09 (0.65, 6.65)	…

Abbreviations: AE, adverse event; CI, confidence interval; OR, odds ratio.

a Bivariate analysis.

b Multivariate analysis; includes only those variables that were associated with AEs in unadjusted bivariate analysis at *P* < .20.

c Significant at *P* < .05.

In multivariate analysis, after controlling for clustering by health facility, circumcisions performed by a provider with 6 or more years of professional experience were *less* likely to be associated with a moderate or severe AE at 7 days (OR = 0.39, 95% CI = 0.17–0.89; *P* < .05) (Table 3). Circumcision clients who were employed (OR = 2.41, 95% CI = 1.06–5.51; *P* < .05) and 18 years and older (OR = 3.75, 95% CI = 1.92–7.33; *P* < .05) were *more* likely to experience a moderate or severe AE at 7 days post-circumcision.

Participants circumcised by providers with 6 or more years of professional experience were less likely to have an adverse event.

### Client Satisfaction With the Circumcision

At 60 days post-circumcision, over 99% of study participants said they were satisfied; 99% reported their partner was satisfied; and over 99% said they would recommend medical circumcision to a friend or family member.

Nearly all men were satisfied with their circumcision.

## DISCUSSION

The intent of this study was to evaluate the safety of trained non-physician clinicians—nurses and clinical officers—performing adult male circumcisions. These clinicians already had experience conducting minor surgeries and were trained on male circumcision techniques until they achieved competency. Men in this study experienced no immediate adverse outcomes associated with the circumcision or with postoperative procedures. Some did present with AEs at 7 days post-circumcision; fewer presented with AEs at 60 days post-circumcision. There was no difference in the rate of moderate and severe adverse events between participants who were circumcised by a nurse or a clinical officer.

Our findings of AE rates of 2.1% and 1.9%, for nurses and clinical officers, respectively, are lower than moderate/severe AE rates (3.8%) reported by Gray et al. in the clinical trial setting.^3^ AE rates in such controlled trials may be higher than those found in observational studies, due to heightened vigilance. However, our findings are also similar to those found in a more typical service-delivery setting with circumcisions performed by trained non-physicians; Herman-Roloff and colleagues reported an AE rate (moderate and severe) of 2.1% in the context of passive surveillance (routine clinical data reported by providers) and 7.5% in the context of active surveillance (outreach by research staff to identify and report events) in Nyanza Province, Kenya.^19^ While our detection of AEs was passive (that is, among participants returning to the clinic), 98% of participants returned for the 7-day follow-up visit. AE rates in this study were thus within an acceptable level compared with data from both the clinical-trial and service-delivery settings.

Adverse event rates for non-physicians in this study were similar to rates reported in typical service-delivery settings.

Our findings are supported by similar studies evaluating the provision of circumcision by non-physician providers. Buwembo and colleagues found no significant differences in moderate or severe AE rates between men whose circumcision was performed by physicians versus clinical officers (1.5% and 0.68%, respectively), after adjusting for other factors.^20^ Herman-Roloff and colleagues found no difference in the odds of experiencing an AE when providers of any type had reported previously performing at least 100 circumcisions.^19^ Others have also found that AE rates decreased as experience increased (based on number of male circumcisions performed).^23^^–^^25^ In our study, having conducted at least 100 circumcisions during the study period did not predict moderate or severe AEs; although participants circumcised by providers with less professional work experience (< 6 years) had increased risk of experiencing moderate or severe AEs.

We also investigated client-related risk factors for AEs, as some of these may be predictors or proxies for clients' behavior and risk post-surgery. Adult men, ages 18–54 years, were at higher risk of experiencing moderate or severe AEs than youth, ages 13–17 years. Adult men were more likely to experience bleeding than young men, which may not be surprising given differences in vascularization in pre-adolescents compared with adults. Adult men were also more likely to experience swelling (but not hematoma). From our data we cannot say why this occurred, and further investigation is warranted. Being employed was a risk factor for experiencing an AE. It is possible that employed men returned to work soon after the procedure, and in so doing disrupted the healing process.

### Limitations and Strengths of the Study

Some limitations should be kept in mind. We did not have data on the number of circumcisions performed by each provider prior to participating in the study. Thus, we cannot evaluate provider proficiency prior to starting the study, although we know that providers were deemed competent at completion of their training. In addition, although we report AEs that we deemed to be causally associated with the circumcision, the rates we found may either overestimate or underestimate truly causal AE rates. We may have been underpowered to detect small significant differences, and results of our multivariate analysis are limited by wide confidence intervals and potentially unstable estimates. Finally, findings with regard to provider skill and experience must be interpreted with caution given the small number of providers who conducted circumcisions in the study. Our findings may not be generalizable to other service-delivery settings.

This study also has several strengths. Our retention rate at the 7-day follow-up visit was exceptionally high, and considerably higher than 7-day follow-up rates in the routine service-delivery setting, which averaged 27.5% from 2008 to 2011,^26^ decreasing the possibility that men returning for follow up were more likely to be those experiencing AEs. Further, as male circumcision is a key preventive measure for HIV, it is imperative to better understand risk factors associated with AEs after the procedure in order to make it as safe as possible. Information on such risk factors is currently limited in the literature.^19^^,^^20^ Our study contributes to a better understanding of these risk factors, and as such provides information that can strengthen male circumcision services. Our study also adds to the current body of evidence regarding task shifting for male circumcision in the routine service-delivery setting, further suggesting that trained nurses and clinical officers can provide safe medical male circumcision, and thereby facilitate increased availability and access to circumcision services.
